# Ectopic Hepatic Tissue Presented as a Posterior Mediastinal Mass

**DOI:** 10.5402/2011/120690

**Published:** 2010-11-25

**Authors:** Eugene Rozen, Gary Stephens, Armand Asarian, Philip Xiao

**Affiliations:** ^1^Department of Surgery, The Brooklyn Hospital Center, NewYork-Presbyterian Healthcare System, Brooklyn, NY 11201, USA; ^2^Department of Pathology, The Brooklyn Hospital Center, NewYork-Presbyterian Healthcare System, Brooklyn, NY 11201, USA

## Abstract

We present a young patient with respiratory complaints that was found to have a mass in her right posterior mediastinum. The mass was diagnosed to be ectopic histologically unremarkable hepatic tissue. We have also reviewed several of the few intrathoracic ectopic liver cases in the literature, along with a brief discussion of the significance of such a finding.

## 1. Presentation

A young patient admitted from the emergency department with complaints of difficulty breathing and shortness of breath. Physical examination was unremarkable, including auscultation, palpation and percussion of her chest. She was ambulatory at admission and awake, alert, and oriented. Her medical history included diabetes, anemia, hyperlipidemia and asthma. Her past surgical history included endometrial ablation and multiple kidney biopsies. She was taking simvastatin, low dose aspirin, lisinopril, metformin, and a number of asthma medications. In the past she had taken antidepressants and antipsychotics. Her labs were mostly within normal limits with the exception of microcytic anemia and slight leukocytosis with slight neutrophilia.

**Figure 1:**
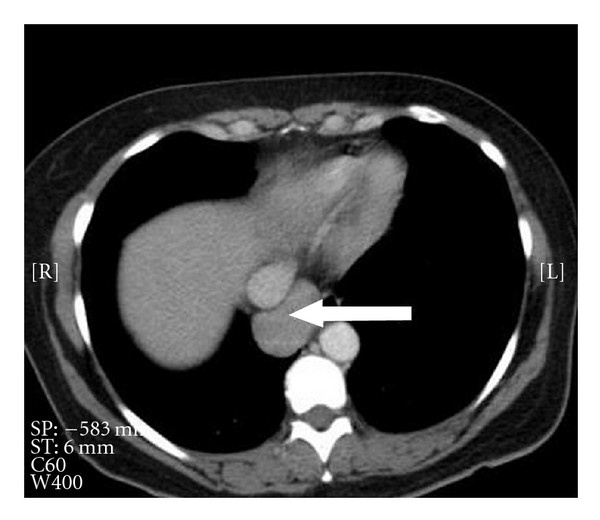
The CT scan showing a 4.5 × 3.3 cm mildly enhancing soft tissue mass in the right posterior mediastinum.

Her symptoms were attributed to her asthma, for which she was treated, but an X-ray and chest CT were ordered. The X-ray was unremarkable except two left sided possible granulomas. The CT scan (Figure 1), in addition to the X-ray findings, showed a 4.5 × 3.3 cm mildly enhancing soft tissue mass in the right posterior mediastinum at the cardiophrenic angle separate from the orthotopic liver. The radiology report suspected an enlarged lymph node or an esophageal mass. She was admitted for further evaluation.

## 2. Surgery

The diagnosis at admission was a benign mediastinal neoplasia, possibly a benign paraganglioma. A video assisted thorocoscopy with possible thoracotomy and excision was consented to by the patient and performed. The mass was well circumscribed and soft and was resected in its totality. The surgeon's finding agreed the CT in seeing no transdiaphragmatic connection between the thoracic mass and the liver. It was noted in the operative record that the mass “appeared to have the consistency of liver”. Two specimens were sent to pathology for further investigation. There were no postoperative complications.

## 3. Pathology

**Figure 2:**
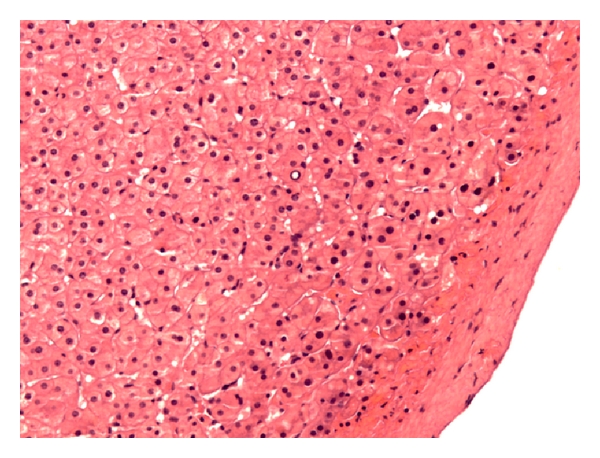
Microscopic examination revealing sheets of benign polygonal cells arranged in three-dimensional plates lined with sinusoids on two sides.

**Figure 3:**
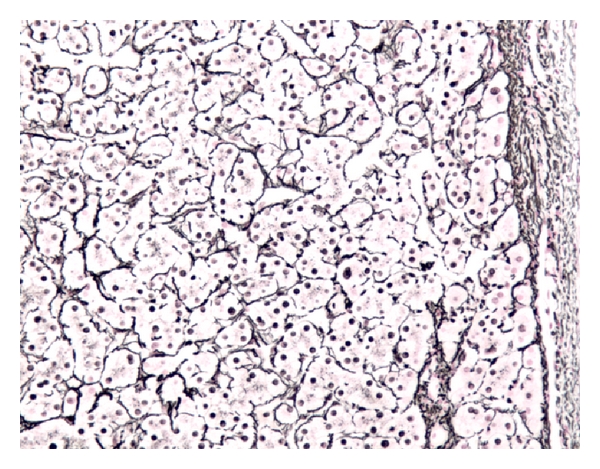
Reticulin stain highlighting the framework of the hepatocytes.

Grossly, the specimens were two fragments of well encapsulated nodule measuring 4 × 3.8 × 3.2 cm in aggregates. Cut section revealed soft, fleshy, dark-brown tissue. Microscopic examination revealed sheets of benign polygonal cells about 25–30 microns, arranged in three dimensional plates that radiated away (Figure 2). The cells themselves were lined by sinusoids on two sides, uniform, with abundant granular and eosinophilic cytoplasm with scattered fat vacuoles, glycogen, and central, round to oval nuclei. Mitotic figures were rare. Bile canaliculi were also present. Immunostaining revealed that the sample was negative for chromogranin A, synaptophysin, neuron-specific enolase and S-100 protein. AE1/AE3 immunostain highlighted the bile ducts, and reticulin stain highlighted the framework of the hepatocyte (Figure 3 In combination, the morphological features and the immunohistochemical profile are characteristic of histologically unremarkable liver tissue. The diagnosis of benign ectopic hepatic tissue was thereby rendered.

## 4. Discussion

Difficulty breathing, the patient's presenting complaint, could indicate abnormalities in any number of thoracic structures. With the results of the CT scan, it became apparent that this symptom was a mass effect in her mediastinum on her lungs. After the surgical excision of the mass of hepatic tissue, there were no further symptoms. 

 Other cases of ectopic liver have been described in the literature, but are uncommon and generally occur in adjacent structures, most often the gall bladder, or elsewhere in the abdomen [1]. Ectopic intrathoracic liver tissue is quite rare [2]. Thoracic hepatic tissue may be a finding with diaphragmatic hernias, either iatrogenic following injury to the diaphragm [3] or congenital in neonates [4], and in one report the ectopic liver was outside of the hernia sac, but connected to the liver [5]. There is a report of multiple liver foci within the lungs in a woman following a heart transplant [6]. There is also a report of the supradiaphragmatic presence of a mass of liver tissue in a cat [7]. 

 Etiologies of ectopic liver are varied. They may be congenital, as the liver and lungs develop embryologically in close proximity [2, 6], but this is unlikely in a middle-aged patient with recent onset of symptoms. They may also result from diaphragmatic hernias or their repair as well as diaphragmatic injury, but there is no reason to believe that this was the case with this patient. The authors of a similar case report speculated that hepatocytes may be hematogenously disseminated and then proliferate in their ectopic destinations, and this is a possibility in our case [6]. The vascular supply of the mass seemed, intraoperatively, to be from the aorta, but due to the absence of any available prior imaging studies, the reason for a sizeable body of hepatic tissue finding itself superior to the diaphragm will have to remain a point of conjecture.

Although the hepatic tissue itself was benign, there is still a need for treatment. Clearly in this case there was a mass effect that produced respiratory symptoms, but could have involved any other mediastinal or thoracic structure leading to symptoms in the gastrointestinal, cardiovascular or neurological systems. There is an additional risk, beyond the mass effect, that the ectopic tissue could undergo a malignant transformation and there have been numerous reports [8, 9] of hepatocellular carcinoma arising from an ectopic focus in the absence of carcinoma in the orthotopic liver, possibly resulting from abnormal vasculature and biliary tracts [9].

 The differential diagnosis for a mediastinal mass includes various tumors (thymoma, teratoma, neurofibroma, etc.), lymphadenopathy (infectious or neoplastic as in Hodgkin disease), vascular aneurysms, cysts and spinal lesions. As this and other cases have shown, ectopic liver, though rare, could be added to this list. Although excisional biopsy was the right choice in this case, there may have been room for further preoperative investigation. The preferred diagnostic method would be endoscopic ultrasound with needle biopsy, although percutaneous fine needle biopsy is also an option. The endoscopic approach has been shown to be effective in investigating mediastinal masses of various origins [10]. Should the mass have been more anterior or superior in the mediastinum, transbronchial biopsy may have been an option. Understanding the etiology would naturally guide treatment. Should the cause of the mass have been found to be reducible with medical treatment, surgery may have been avoided. In our case, of course, preoperative elucidation of the disease process would not have changed the treatment.

Mebrophenin scintigraphy can localize hepatic tissue rather specifically, and could create a clear and non-invasive view of the ectopic liver, as well as highlight any other foci of hepatic tissue than may need to be addressed [11]. Obviously, this would be an unreasonable diagnostic modality to employ, but if the nature of the mass was known, it could have been used to discover or track other sites of ectopic hepatic growth.

## References

[1] Triantafyllidis I, Papapavlou L, Nikoloudis N (2009). Ectopic liver tissue attached to the gallbladder wall: a case report. *Cases Journal*.

[2] Babu R, van der Avoirt A (2001). Ectopic intrathoracic liver. *Pediatric Surgery International*.

[3] Yoshino I, Yamaguchi M, Kameyama T (2006). Extension of liver tissue into the thorax following a right extrapleural pneumonectomy for malignant pleural mesothelioma. *Annals of Thoracic and Cardiovascular Surgery*.

[4] Luoma R, Raboei E (2003). Supradiaphragmatic accessory liver: a rare cause of respiratory distress in a neonate. *Journal of Pediatric Surgery*.

[5] Huang CS, Hsu WH, Hsia CY (2007). Supradiaphragmatic ectopic liver: delayed traumatic hepatic hernia mimics pulmonary tumor. *Thoracic and Cardiovascular Surgeon*.

[6] Mehta RI, Lai CK, Kee S, Fishbein MC (2010). Intrapulmonary ectopic liver after orthotopic heart transplantation. *Archives of Pathology and Laboratory Medicine*.

[7] Dhaliwal RS, Lacey JK (2009). *Ectopic* hepatic parenchyma attached to the diaphragm: simulating a pulmonary mass in a cat. *Journal of the American Animal Hospital Association*.

[8] Seo UH, Lee HJ, Ryu WS (2008). Laparoscopic resection of a hepatocellular carcinoma arising from an ectopic liver. *Surgical Laparoscopy, Endoscopy and Percutaneous Techniques*.

[9] Arakawa M, Kimura Y, Sakata K, Kubo Y, Fukushima T, Okuda K (1999). Propensity of ectopic liver to hepatocarcinogenesis: case reports and a review of the literature. *Hepatology*.

[10] LeBlanc JK Endoscopic ultrasound-guided fine-needle aspiration in the mediastinum. http://www.uptodate.com/.

[11] Díeza MJ, Canelaa T, Villasa A, Gil M (2009). Contribution of 99mTc-mebrofenin scintigraphy to the diagnosis of ectopic liver: a case report. *Revista Española de Medicina Nuclear*.

